# An update on attachment systems for mandibular implant overdentures: A review

**DOI:** 10.6026/9732063002001623

**Published:** 2024-11-30

**Authors:** Charushila Sardar, Gaurang Mistry, Nimrat Kaur Randhawa, Padmapriya Puppala, Ankita Chitnis, Sanpreet Singh Sachdev

**Affiliations:** 1 Department of Prosthodontics, D.Y. Patil Deemed to be University, School of Dentistry, Navi Mumbai, Maharashtra, India; 2 Department of Oral Pathology and Microbiology, Bharati Vidyapeeth (Deemed to be University) Dental College and Hospital, Navi Mumbai, Maharashtra, India

**Keywords:** Hypochlorous acid, chronic wound, infection, co-morbidities

## Abstract

The present systematic review aims to evaluate the global use of attachment systems for mandibular implant overdentures, focusing on their impact on clinical outcomes, patient satisfaction and prosthesis stability. Mandibular overdentures supported by implants have become a standard of care for edentulous patients, with various attachment systems such as bar, ball and locators being commonly utilized. The review synthesizes findings from numerous studies, examining differences in retention, ease of maintenance, cost-effectiveness and long-term success rates across different attachment modalities. The analysis also delves into patient-centered outcomes, including comfort, ease of use and quality of life, providing a comprehensive overview of the efficacy of each system. By comparing and contrasting these systems, the review identifies trends and preferences in clinical practice, highlighting the benefits and drawbacks of each approach. The findings indicate that while all systems show promise in enhancing the functionality of mandibular overdentures, patient-specific factors and clinician preferences play crucial roles in the selection process.

## Background:

The lack of retention, support and stability in conventional complete dentures often leads to significant difficulties for edentulous individuals, resulting in diminished chewing capacity and overall oral function. For completely edentulous patients, treatment options include either a full denture or an implant-supported prosthesis. Among these, mandibular implant overdentures have been shown to significantly improve masticatory function and patient satisfaction, offering a preferable alternative for many [1]. The attachment system used in mandibular implant overdentures plays a crucial role in enhancing denture stability and retention, which in turn influences the success of the prosthesis and the quality of life for the patient [2]. Over the years, various attachment systems have been developed to improve the functionality of mandibular implant overdentures. The most commonly used systems include bar, ball and magnetic attachments, as well as other mechanical devices that provide retention and stability [3]. These systems are fundamental to the success of mandibular implant overdentures, which represent a transformative advancement in the field of prosthodontics. For countless edentulous and partially edentulous individuals worldwide, these removable dental prostheses supported by implants have dramatically improved their oral health and quality of life.

Technological advances, improved materials and growing clinical experience have all contributed to the evolution of attachment systems over time [4]. This review aims to explore the development and global application of attachment systems for mandibular implant overdentures, examining their progression from early designs to contemporary solutions that offer superior outcomes. The early history of implant overdentures, dating back to the early 1900s, saw the use of rudimentary attachment methods, primarily ball attachments or bar-and-clip systems [5]. These early systems, while innovative for their time, had limitations in terms of hygiene, maintenance and long-term performance, prompting the search for improved alternatives. One of the most significant advancements in attachment systems was the introduction of Locator attachments in the late 20th century. These attachments offered a self-aligning design that greatly enhanced ease of use, stability and retention, addressing many of the shortcomings of earlier systems. Similarly, magnetic attachments have provided a viable alternative, particularly in regions where their simplicity and durability are highly valued [6].

Advances in materials and manufacturing, such as the use of CAD/CAM technology, have further refined these systems, allowing for more precise, patient-specific solutions that improve the overall performance of mandibular implant overdentures. Globally, the development and adoption of attachment systems have been influenced by various factors, including patient demographics, clinical preferences and regional regulatory environments. For example, in North America, Locator attachments have gained popularity due to their user-friendliness and ability to meet patient expectations for secure and comfortable overdentures [7]. In Europe, a wider range of systems, including ball and stud attachments, are commonly used, reflecting the region's role as a hub for dental implant innovation. In Asia, magnetic attachments have become particularly popular, while South America has seen diverse adoption patterns, with different systems being selected based on individual patient needs and clinician preferences [8].

Despite these advancements, the field of mandibular implant overdenture attachment systems continues to face challenges. The integration of digital dentistry, including CAD/CAM technology, is one of the leading trends, enabling more precise and personalized treatment outcomes. Immediate loading protocols and the growing demand for improved aesthetics and patient comfort are also driving innovation in attachment systems [9]. Long-term durability and maintenance remain key concerns, with on-going research focused on developing materials and designs that can withstand the rigors of long-term use. As we look to the future, the potential for further innovation in mandibular implant overdenture attachment systems is vast.

Developments in biocompatible materials, enhanced osseointegration and minimally invasive surgical techniques are likely to yield more patient-specific, durable and aesthetically pleasing solutions. Additionally, the integration of artificial intelligence and predictive modelling could further refine the customization of attachment systems, leading to improved outcomes for patients worldwide [10]. A previous systematic review by Kim *et al.* in 2012 had reported that implant survival rate of mandibular overdentures is high regardless attachment systems [17]. However, it is essential to continually update the literature with new studies given the evolution of prosthesis and techniques over time. Thus, it was essential to effectively bridge the gap in literature. This systematic review aims to provide a comprehensive global perspective on the current state of attachment systems for mandibular implant overdentures, offering insights that can guide future research and clinical practice.

## Methods and Materials:

## Formulation of the research question:

This systematic review was guided by the PICOS criteria to formulate a focused research question. The elements were defined as follows:

[1] Participants/Population: Edentulous patients with mandibular overdentures, aged between 18 to 75 years.

[2] Interventions: The use of mandibular overdentures.

[3] Comparators/Control: Different attachment types used in mandibular overdentures.

[4] Study types: Randomized controlled trials (RCTs), case-control studies and retrospective studies that meet eligibility criteria, published in English, with accessible full texts.

[5] Context: The review included studies from high, low and middle-income countries, considering various systems and loading techniques, with outcomes compared across sub-groups derived from data extraction.

[6] Main outcomes: Comparison of retention across different attachment types, focusing on implant and prosthesis survival rates and biological and prosthesis complications.

[7] Additional outcomes: Treatment prognosis based on age, implant placement duration and attachment types used.

The research question formulated was: "Are there any differences in prosthodontic complications, retention, peri-implant tissue alterations and patient satisfaction with different implant overdenture attachment systems in totally and partially edentulous mandibular arch rehabilitation with late implant placement?"

## Search strategy:

## Eligibility criteria for studies:

## Inclusion criteria:

[1] Participants with a completely edentulous mandibular arch aged 18 to 75 years.

[2] Studies published between in the past decade from 2015 to 2024.

[3] Studies evaluating the effectiveness of two or more implant-supported overdentures with a minimum one-year follow-up.

[4] Outcome measures including implant-supported overdenture life, denture stability, bone loss and utility.

[5] Studies on late implant placement with delayed loading protocol, overdentures retained with two or more interforaminal implants and attachments on root-form endosseous implants.

[6] Randomized controlled trials and clinical studies conducted *in vivo* or *ex vivo.*

[7] Articles published in English or with available English translations.

## Exclusion Criteria:

[1] Conference proceedings, personal communications, letters to editors, case reports and other un-peer-reviewed literature.

[2] Studies focusing on maxillary arch overdentures.

[3] Case reports without proper statistical comparisons and studies involving patients above 75 years.

## Search terms and databases:

The search was conducted across four electronic databases: PubMed, Google Scholar, ScienceDirect and SCOPUS, using Boolean operators "AND" and "OR." The search terms included "implant-supported overdenture," "mandibular overdenture," "overdenture attachment systems," and related synonyms. MeSH terms and proximity operators were also employed. The search strategy and details were summarized in a PRISMA flowchart and Table 1. Additionally, key journals like the Journal of Prosthetic Dentistry and Journal of Oral Rehabilitation were manually searched.

## Study selection:

Two independent reviewers screened the articles using Rayyan software. Initial screening of titles was followed by abstracts and eligible studies were further assessed for conflicts. A third reviewer resolved any disagreements, ensuring consistency in study inclusion.

## Data extraction:

Data were extracted using a structured Microsoft® Excel® format, including details such as author, publication year, study type, sample size, outcomes and final inferences. Two reviewers independently performed the extraction, overseen by a third reviewer to ensure data quality and resolve any discrepancies.

## Quality assessment:

The Joanna Briggs Institute (JBI) Quality Assessment Tool was used to evaluate the selected studies. This involved critical appraisal by two independent reviewers, with a third resolving any issues. The NIH criteria for randomized controlled trials were also applied. Risk of bias was assessed at the study and outcome levels using the JBI tool, covering potential biases in selection, performance, detection, attrition and reporting.

## Registration:

The systematic review was registered under the PROSPERO framework.

## Results:

## Study characteristics:

We included six studies, five of which were randomized controlled trials and one was a retrospective study [11, 12, 13, 14, 15-16]. The selection process of studies from search until final inclusion in the data qualitative synthesis is depicted in Figure 1 (see PDF). Three studies were conducted in 2021 and two in 2019. Two of the studies did not mention the implant brand used in the study; the remaining three studies provided the details of the implant brand used. One of the studies did not mention the type of attachment system; while the other four studies mentioned it. The studies reported similar outcomes (Table 1). The quality assessment of the studies according to the NIH criteria is shown in Table 2.

## Discussion:

The findings of this systematic review highlight the complex interplay between attachment systems used in mandibular implant over-dentures and their impact on clinical outcomes. The diversity of attachment systems, ranging from ball and bar to Locator and magnetic attachments, reflects the on-going efforts to enhance prosthodontic solutions for edentulous patients. These systems have been extensively studied, with varying results concerning retention, patient satisfaction and biological complications. Retention remains a critical factor in the success of mandibular implant over-dentures.

The studies reviewed indicate that different attachment systems offer distinct advantages in terms of retention and stability. For instance, Resende *et al.* [11] and Albuquerque *et al.* [16] demonstrated that ball attachments provided superior retention in the early stages post-implantation, which significantly contributed to patient satisfaction. On the other hand, Patil *et al.* [13] highlighted that Locator attachments, while initially less retentive than ball attachments, offered a balance between retention and ease of maintenance over time. These findings suggest that while ball attachments may be more suitable for patients requiring immediate retention, Locator attachments might be preferable for long-term use. Patient satisfaction is closely linked to the comfort and functionality of the overdenture, both of which are influenced by the attachment system used. Abdel *et al.* [12] found that ball attachments provided better initial patient satisfaction compared to CM-LOC attachments, primarily due to their higher initial retention. However, the long-term satisfaction reported by Patil *et al.* [13] with Locator attachments suggests that as patients become accustomed to the prosthesis, factors such as ease of cleaning and maintenance play a more significant role in their overall satisfaction. This indicates that while initial retention is important, the long-term usability of the attachment system is crucial for sustained patient satisfaction.

The impact of attachment systems on peri-implant tissue health and biological complications is another crucial consideration. Studies by Resende *et al.* [11] and Enkling *et al.* [15] reported that specific attachment systems, particularly those with high retention forces, were associated with increased soft tissue complications, such as mucosal irritation and inflammation. This finding underscores the importance of balancing retention with the preservation of peri-implant tissue health. Moreover, the review by Ortensi *et al.* [14] suggested that splinted attachment systems might reduce the incidence of these complications by distributing occlusal forces more evenly, thereby protecting the peri-implant tissues. The durability and maintenance requirements of the attachment systems are also critical for the long-term success of mandibular implant overdentures. Albuquerque *et al.* [16] highlighted that retention with certain attachment systems, such as ball attachments, tended to decrease over time, necessitating periodic maintenance. Conversely, the Locator system, as observed by Patil *et al.* [13], offered a more consistent retention profile, which could potentially reduce the need for frequent adjustments and replacements. This aspect is particularly important in clinical settings where access to regular follow-up care may be limited.

The review also revealed notable regional variations in the adoption and success of different attachment systems. For example, magnetic attachments have gained popularity in Asia due to their simplicity and lower maintenance requirements, as discussed by several studies in this review. In contrast, the use of locator attachments is more prevalent in North America, where patient preferences for ease of use and maintenance are emphasized [7]. These regional differences highlight the importance of considering local clinical practices, patient demographics and access to care when selecting an attachment system. Despite the valuable insights gained from this review, several limitations must be acknowledged. The heterogeneity of the studies, in terms of design, sample size and follow-up duration, complicates direct comparisons between attachment systems. Additionally, the lack of standardized outcome measures across studies limits the ability to draw definitive conclusions. Future research should focus on long-term randomized controlled trials with standardized protocols to better understand the relative advantages of different attachment systems.

## Conclusion:

The choice of attachment system for mandibular implant over-dentures should be tailored to the individual patient's needs, considering factors such as retention, ease of maintenance and peri-implant tissue health. The findings of this review underscore the importance of a balanced approach that prioritizes both prosthetic functionality and patient well-being. Further research is needed to explore emerging technologies and materials that could enhance the performance and longevity of these attachment systems.

## Figures and Tables

**Table 1:** Studies included in the review

**Author**	**Year**	**Study Type**	**Sample Size**	**Groups**	**Implant Type**	**Attachment System**	**Population**	**Outcome**
Resende *et al.* [11]	2021	Randomized Controlled Trial	1-IOD: 23; 2-IOD: 24	2 groups (Single-implant, Double-implant)	Tissue level Straumann® Standard Plus SLActive®	Fully edentulous subjects	1-IOD group: lower width of keratinized tissue (p = 0.040); 2-IOD group: lower lingual mucosa thickness (p = 0.026)	
AbdelAal *et al.* [12]	2019	Randomized Controlled Trial	Ball: 31; CM-LOC: 34	Ball vs. CM-LOC	Zimmer Dental, 3.7 mm diameter, 10 mm length	Ball or CM-LOC	Fully edentulous subjects	Ball attachment: Better results 2 weeks post-pickup due to high initial retention compared to CM-LOC
Patil *et al.* [13]	2021	Randomized Controlled Trial	Single: 26; Two: 26	Single implant vs. Two implants	Roxolid SLActive® (Straumann) 3.3 mm or 4.1 mm diameter, 10 mm or 12 mm length	LOCATOR® (Zest Anchors)	Completely edentulous	Improved QoL in elderly edentulous Malaysian patients at 1 month of immediate loading and 1 year of recall
Ortensi *et al.* [14]	2019	Retrospective Study	46	Mandibular: 27 (Splinted and Unsplinted)	Not specified	Ball attachment	Completely edentulous	High implant/prosthetic success, low mechanical/biological complications, high patient satisfaction
Enkling *et al.* [15]	2021	Randomized Controlled Trial	12	Not specified	SIC ace (SIC invent AG), 6 mm length, 4.0 or 4.5 mm diameter	Retentive ball vs. non-retentive dome	Completely edentulous	6 mm short implants viable at mandibular molar sites; patients prefer ball abutment over dome
Albuquerque *et al.* [16]	2018	Randomized Controlled Trial	24	Not specified	Not specified	Cylindrical (LA) vs. Ball (RA)	Completely edentulous	Higher overall retention for RA (p = 0.0005); retention declines over time (P < 0.0001)

**Table 2:** Quality assessment of the studies

	**NIH Criteria for assessing clinical studies**					
	**Resende *et al.***	**Abdel Aal*et al.***	**Patil *et al.***	**Ortensi *et al.***	**Enkling *et al.***	**Albiquerque *et al.***
1. Was the study described as randomized, a randomized trial, a randomized clinical trial, or an RCT?	Yes	Yes	Yes	x	Yes	Yes
2. Was the method of randomization adequate (*i.e.,* use of randomly generated assignment)?	NA	NA	NA	x	Yes	Yes
3. Was the treatment allocation concealed (so that assignments could not be predicted)?	Yes	Yes	Yes	x	Yes	Yes
4. Were study participants and providers blinded to treatment group assignment?	Yes	Yes	Yes	NA	Yes	Yes
5. Were the people assessing the outcomes blinded to the participants' group assignments?	Yes	Yes	Yes	NA	Yes	Yes
6. Were the groups similar at baseline on important characteristics that could affect outcomes (*e.g.,* demographics, risk factors, co-morbid conditions)?	Yes	Yes	Yes	Yes	Yes	Yes
7. Was the overall drop-out rate from the study at endpoint 20% or lower of the number allocated to treatment?	Yes	Yes	Yes	Yes	Yes	Yes
8. Was the differential drop-out rate (between treatment groups) at endpoint 15 percentage points or lower?	Yes	Yes	Yes	Yes	Yes	Yes
9. Was there high adherence to the intervention protocols for each treatment group?	Yes	Yes	Yes	Yes	Yes	Yes
10. Were other interventions avoided or similar in the groups (*e.g.,* similar background treatments)?	x	x	x	x	x	x
11. Were outcomes assessed using valid and reliable measures, implemented consistently across all study participants?	Yes	Yes	Yes	Yes	Yes	Yes
12. Did the authors report that the sample size was sufficiently large to be able to detect a difference in the main outcome between groups with at least 80% power?	Yes	Yes	Yes	Yes	Yes	Yes
13. Were outcomes reported or subgroups analyzed prespecified (*i.e.,* identified before analyses were conducted)?	Yes	Yes	Yes	Yes	Yes	Yes
14. Were all randomized participants analyzed in the group to which they were originally assigned, *i.e.,* did they use an intention-to-treat analysis?	x	x	x	x	x	x
